# Spontaneous Migration of a Postcholecystectomy Surgical Clip From the Common Bile Duct to the Cecum Nineteen Years After Surgery: A Case Report

**DOI:** 10.1155/crgm/7768285

**Published:** 2026-08-20

**Authors:** Marina Takawy, Tausif Syed, Mostafa Najim, Richard Alweis, Jason Gutman

**Affiliations:** ^1^ Department of Medicine, Rochester Regional Health-Unity Hospital, Rochester 14626, New York, USA, duhs.edu.pk; ^2^ Division of Gastroenterology, Department of Medicine, Rochester General Hospital, Rochester 14621, New York, USA, rochesterregional.org

**Keywords:** case report, cholecystectomy, clip migration, obstructive jaundice, spontaneous resolution

## Abstract

Postcholecystectomy clip migration (PCCM) is a rare but recognized complication following cholecystectomy. The clinical presentation of PCCM often mimics that of choledocholithiasis, with symptoms including right upper quadrant pain and obstructive jaundice. Although the exact mechanisms underlying clip migration remain unclear, several theories have been proposed in the literature. While most cases occur shortly after the procedure, with clips migrating to the common bile duct (CBD) or, less commonly, to the duodenum; some instances may occasionally present much later, such as the case described here. An 85‐year‐old female presented with symptoms consistent with CBD obstruction, and the initial imaging revealed a clip in the CBD. Endoscopic retrograde cholangiopancreatography (ERCP) was planned; however, subsequent imaging showed that the clip had disappeared. Three days later, the clip was found in the cecum, and the patient’s symptoms had improved. Given the patient’s clinical progress, conservative management was pursued, allowing the clip to pass naturally without further intervention. While clip migration can lead to serious complications such as CBD obstruction or cholangitis, requiring procedural or surgical management, spontaneous resolution with conservative care, although rare, has been reported. The factors contributing to clip migration may include procedural factors early after surgery or chronic inflammatory changes and tissue necrosis that develop years after the cholecystectomy. Emerging techniques such as clipless surgery and the use of absorbable sutures are under development to reduce the risk of clip migration. In conclusion, despite its rarity, PCCM should be considered in patients with a history of cholecystectomy who present with symptoms suggestive of CBD obstruction. Although uncommon, clip migration may occur without significant complications and can resolve spontaneously, as demonstrated in this case. Conservative management may be an appropriate approach in select cases, particularly when the patient is clinically improving.

## 1. Introduction

Cholecystectomy is one of the most commonly performed surgical procedures and is generally well tolerated, with an overall complication rate of 3%–10%. Laparoscopic cholecystectomy replaced the open approach as the preferred surgical management for gallstone disease in the early 1990s [1, 2]. Early complications of cholecystectomy include biliary, bowel and vascular injury, and infections; while late complications include biliary stricture, fistula, and stones. Postcholecystectomy clip migration (PCCM) is a rare complication of cholecystectomy, with significant morbidity, but no reported mortality [3]. PCCM has been reported from 11 days to 20 years after cholecystectomy, with a median interval of approximately 26 months [4, 5]. The majority of PCCM cases documented in the literature involved clip migration to the common bile duct (CBD), and less commonly to the duodenum, within the first 10 years after the procedure [3, 4, 6]. The management of these cases typically involved endoscopic retrograde cholangiopancreatography (ERCP) and sphincterotomy [7]. Here, we present a unique case of uncomplicated clip migration to the cecum 19 years after cholecystectomy, managed conservatively.

## 2. Case Description

An 85‐year‐old female, with a history of hypertension, hyperlipidemia, coronary artery disease, stroke, appendectomy in the 1950s, and cholecystectomy in 2005, presented to the emergency department with abdominal pain and nausea.

She described midsternal and epigastric pain, rated as 8/10 in intensity; the pain was described as aching/stabbing and waxing and waning in nature for about 30 min. She reported similar episodes exacerbated by food and relieved spontaneously over the last few months. Although the patient was vitally stable on presentation, she subsequently developed chills, rigors, and one episode of bilious emesis while in the emergency department. Physical examination was notable for right upper quadrant and epigastric tenderness. No obvious surgical scars were identified on abdominal examination, and attempts to retrieve the cholecystectomy operative report were unsuccessful precluding a definitive determination of the surgical approach. Significant laboratory findings on the day of admission included newly elevated liver function tests (LFTs) and a normal leukocyte count (see Table 1).

**TABLE 1:** Relevant laboratory results.

Laboratory test	On admission	Day 1	Day 2	Day 5 (on discharge)	Normal reference range
AST (U/L)	218	499	132	22	7–37
ALT (U/L)	140	569	292	58	10–49
Alkaline phosphatase (U/L)	144	185	142	126	46–116
White cell count (× 10^3^) UL	7.5	10.5	6.2	7.3	4–11
Bilirubin, total (mg/dL)	1.0	2.2	1.8	0.4	0.3–1.2

**FIGURE 1:**
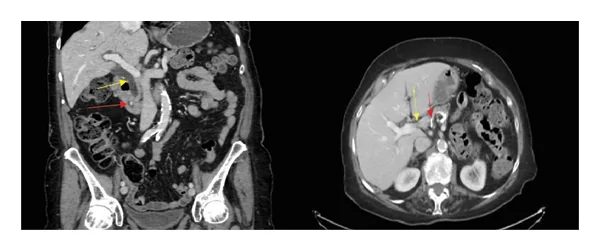
Computed tomography (coronal and axial views) on admission showing a clip obstructing the distal common bile duct (red arrows) and interval worsening of dilation of the common bile duct (up to 15 mm) and intrahepatic biliary ducts (yellow arrows).

CT on admission (Figure 1) revealed interval worsening of dilation of the CBD (up to 1.5 cm) compared to CBD dilatation of 1.1 cm 5 months earlier during another episode of abdominal pain and intrahepatic biliary ducts dilation. Initial management included intravenous fluid resuscitation, analgesia with acetaminophen, and serial monitoring of LFTs and abdominal symptoms. Antibiotics were not administered as there was no evidence of infection.

On Day 1, the gastroenterology service was consulted and per their recommendations, MRCP followed by ERCP were planned the following day for the patient.

**FIGURE 2:**
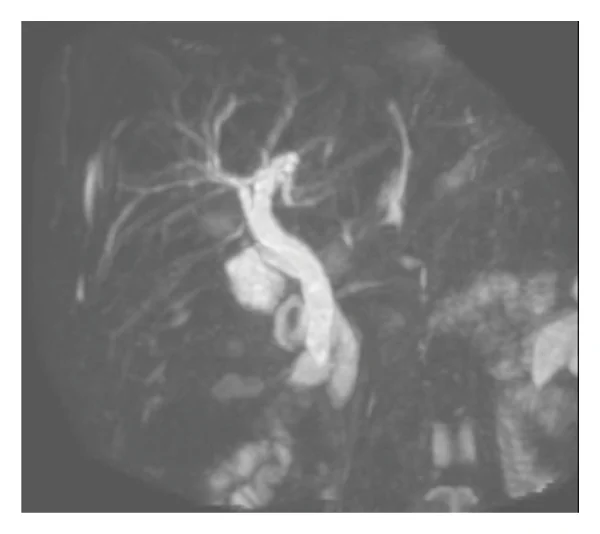
MRCP abdomen demonstrating intra‐ and extrahepatic biliary ductal dilatation. The extrahepatic bile duct measures 13 mm. There is no choledocholithiasis.

MRCP on Day 2 (Figure 2) revealed biliary ductal dilatation, but no clip was detected, suggesting that it had migrated to the duodenal opening. Consequently, the decision was made to delay ERCP in favor of clinical monitoring and conservative management. This approach was chosen because the cause of the biliary obstruction remained unidentified, LFTs were improving, and the risks of ERCP outweighed the benefits given the patient’s age.

**FIGURE 3:**
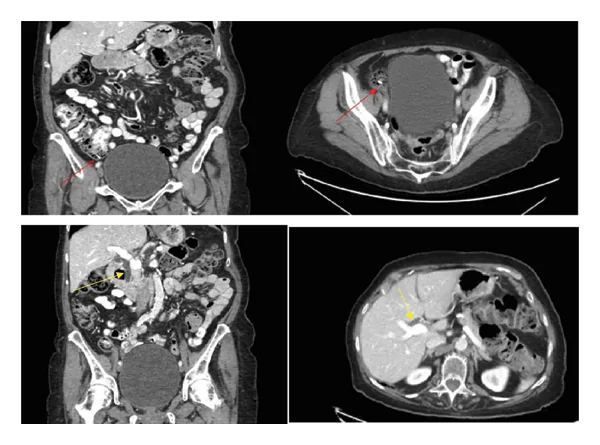
Computed tomography (coronal and axial views demonstrating clip location and CBD) 3 days after admission showing CBD dilation to approximately 10 mm (yellow arrows); previous intrahepatic bile duct distention is essentially resolved. The previously seen surgical clip in the region of the distal common bile duct is no longer evident, and there is a new clip in the region of the cecum (red arrows), presumably the migrated clip.

A repeat CT on Day 3 (Figure 3) demonstrated improvement in CBD dilation to approximately 10 mm, and the previously noted intrahepatic bile duct dilation has essentially resolved. The previously seen surgical clip in the region of the distal CBD is no longer evident, and there is a new clip in the cecum, presumably the migrated clip.

This was accompanied by clinical improvement of the patient and normalization of her laboratory values as shown in Table 1. Consequently, the patient was discharged home in a stable condition. Subsequent follow‐ups with the primary care and gastroenterology clinics did not reveal any recurrence of abdominal pain or hospitalization with similar presentation.

## 3. Discussion

Surgical clips have been widely used over the past decade for both open and laparoscopic cholecystectomy. Walker et al. first reported stone formation around a migrated surgical clip following open cholecystectomy in 1979 [8]. After which, Raoul et al. reported the first case of clip migration after laparoscopic cholecystectomy in 1992 [9]. Fewer than one hundred cases of clip migration are documented in the literature, most of them involving CBD migration occurring within the first 10 years after the procedure [3, 4].

Less than 20 cases of PCCM to the duodenum were identified, most presenting with complications such as bleeding or ulceration, which require immediate interventions [6]. These cases were managed with ERCP and sphincterotomy; surgery was reserved for cases where ERCP failed [7]. To the best of our knowledge, only one case of uncomplicated PCCM to the duodenum with conservative management till spontaneous resolution was identified [10].

Early clip migration is typically related to procedural factors such as imprecise clip placement on the cystic duct stump, leading to incomplete closure of the duct and development of bilioma; bile leakage intraoperatively leading to tissue erosion with subsequent inflammation and adhesions; and the use of more than four endoclips [11–13].

Late migration is believed to result from chronic inflammatory processes induced by the clip or a foreign body response [4, 10, 14]. Another possible explanation proposed by Kitamura et al. involves hepatic compression on the cystic duct and clips leading to their inversion into the CBD, which leads to necrosis of the tissue surrounding the clip and slippage into the CBD [15]. Consistent with the foreign body response theory [4, 10, 14], the late migration observed in this patient (≥ 18 years postsurgery) is likely the result of progressive chronic inflammation and tissue remodeling around the clip, ultimately leading to its detachment and migration. The absence of evidence of acute infection or biliary leakage in the patient supports this chronic indolent process.

Migrated clips may directly obstruct the CBD or serve as a nidus for biliary stone formation. Consequently, the most commonly reported presentation of PCCM resembles choledocholithiasis, potentially leading to further complications such as cholangitis [5]. Other less commonly reported complications in literature include acute pancreatitis, duodenal ulcer, biliary‐colonic fistula, and subdiaphragmatic abscess [10, 14].

Right upper quadrant ultrasound, CT, or MRCP can establish the diagnosis. ERCP remains the gold standard for clip retrieval, with a success rate of 85% [3, 5]. The presence of anatomically challenging cases such as fistulas and strictures or large stones may lead to unsuccessful ERCP; in such cases, open surgery is performed [3]. Novel strategies have been proposed to mitigate the risk of clip migration including absorbable sutures and clipless cholecystectomy using harmonic scalpel or ultrasound shear dissection of the cystic artery and duct [16–21].

## 4. Limitations

The limitations in our case were that since the clip was not retrieved, histopathological examination was not possible. In addition, our records provide no data about the approach of the cholecystectomy procedure in 2005: open or laparoscopic, and no scars were documented on abdominal examination. Laparoscopic surgery and open surgery may differ in clip placement techniques or clip types, and this uncertainty may affect the accurate inference of migration mechanisms.

## 5. Conclusion

Late PCCM migration is a rare complication; however, it should still be kept in the differential of a patient presenting with RUQ pain many years following cholecystectomy.

A conservative management strategy may be appropriate in certain carefully selected cases, particularly when the patient shows clinical improvement with resolving biliary obstruction and the clip has migrated distally (e.g., duodenum or cecum).

## Author Contributions

Marina Takawy, Mostafa Najim, and Tausif Syed: manuscript writing; Richard Alweis and Jason Gutman: manuscript editing and revision.

## Funding

The authors have nothing to report.

## Disclosure

All the authors reviewed and approved the final draft of the manuscript.

This case has been accepted as a poster presentation at the ACG conference 2025, Phoenix, AZ, poster ID P2384.

## Consent

The authors have nothing to report.

## Conflicts of Interest

The authors declare no conflicts of interest.

## Data Availability

The data that support the findings of this study are available from the corresponding author upon reasonable request.
